# Assessment of coronary vascular function with cardiac PET in relation to serum uric acid

**DOI:** 10.1371/journal.pone.0192788

**Published:** 2018-02-13

**Authors:** Seoyoung C. Kim, Nishant R. Shah, James R. Rogers, Courtney F. Bibbo, Marcelo F. Di Carli, Daniel H. Solomon

**Affiliations:** 1 Division of Pharmacoepidemiology and Pharmacoeconomics, Department of Medicine, Brigham and Women’s Hospital, Harvard Medical School, Boston, Massachusetts, United States of America; 2 Division of Rheumatology, Immunology, and Allergy, Department of Medicine, Brigham and Women’s Hospital, Harvard Medical School, Boston, Massachusetts, United States of America; 3 Noninvasive Cardiovascular Imaging Program, Heart and Vascular Institute, Division of Cardiovascular Medicine, Department of Medicine, Division of Nuclear Medicine and Molecular Imaging, Department of Radiology, Brigham and Women’s Hospital, Harvard Medical School, Boston, Massachusetts, United States of America; 4 Lifespan Cardiovascular Institute, Division of Cardiovascular Medicine, Department of Medicine, Brown University Alpert School of Medicine, Providence, Rhode Island, United States of America; Fondazione Toscana Gabriele Monasterio, ITALY

## Abstract

**Background:**

Elevated serum uric acid (SUA) levels have been independently associated with cardiovascular disease. Stress myocardial perfusion positron emission tomography (PET) allows for measurement of absolute myocardial blood flow (MBF) and quantification of global left ventricular coronary flow reserve (CFR). A CFR <2.0 is considered impaired coronary vascular function, and it is associated with increased cardiovascular risk. We evaluated the relationship between SUA and PET-measured markers of coronary vascular function.

**Methods:**

We studied adults undergoing a stress myocardial perfusion PET on clinical grounds (1/2006-3/2014) who also had ≥1 SUA measurement within 180 days from the PET date. Multivariable linear regression estimated the association between SUA and PET-derived MBF and CFR. We also stratified analyses by diabetes status.

**Results:**

We included 382 patients with mean (SD) age of 68.4 (12.4) years and mean (SD) SUA level of 7.2 (2.6) mg/dl. 36% were female and 29% had gout. Median [IQR] CFR was reduced at 1.6 [1.2, 2.0] and median [IQR] stress MBF was 1.5 [1.1, 2.1] ml/min/g. In the adjusted analysis, SUA was inversely associated with stress MBF (β = -0.14, p = 0.01) but not with CFR. Among patients without diabetes (n = 215), SUA had a negative association with CFR (β = -0.15, p = 0.02) and stress MBF (β = -0.19, p = 0.01) adjusting for age, sex, extent of myocardial scar and ischemia, serum creatinine and gout. In diabetic patients (n = 167), SUA was not associated with either CFR or MBF.

**Conclusions:**

In this cross-sectional study, higher SUA is modestly associated with worse CFR and stress MBF among patients without diabetes.

## Introduction

A number of large epidemiologic studies have demonstrated an independent association between serum uric acid (SUA) levels and risk of myocardial infarction (MI), heart failure, stroke, and cardiovascular mortality.[1–3] Elevated SUA is hypothesized to cause increased oxidative stress, microinflammation, lipid oxidation, and inhibition of nitric oxide production. In turn, these all contribute to endothelial dysfunction, microvascular ischemia, interstitial fibrosis, and myocardial dysfunction.[4–6]

Coronary flow reserve (CFR)—the ratio of peak hyperemic myocardial blood flow (MBF) over MBF at rest as measured by positron emission tomography (PET)–is a robust and reproducible clinical measure of the integrated hemodynamic effects of epicardial coronary artery stenoses, diffuse atherosclerosis, vessel remodeling, endothelial dysfunction, and microvascular dysfunction on myocardial tissue perfusion.[7,8] A reduced CFR can be a sign of flow-limiting coronary artery stenoses as it is associated with the overall extent and severity of angiographic coronary artery disease (CAD).[9] Furthermore, a reduced CFR can indicate the presence of coronary vascular dysfunction involving smaller vessels, which increases the severity of inducible myocardial ischemia and sub-clinical myocardial injury beyond the effects of upstream coronary obstruction.[10] Importantly, there is growing, consistent evidence that impaired CFR is independently and incrementally associated with risk for MI, heart failure as well as cardiovascular death.[7,8,11,12]

Over the past decades, the association between hyperuricemia and cardiovascular disease has been extensively studied. The association between SUA and increased cardiovascular risk appears to be only partially accounted by traditional coronary risk factors. This suggests that other mechanisms may contribute to the association between the SUA and increased cardiovascular risk. One such mechanism may involve the potential adverse effect of SUA on vascular function and in particular endothelial function, thereby increasing the potential for coronary vasoconstriction and thrombosis. To date, the direct effect of hyperuricemia on coronary vascular function, as assessed by CFR, has not been studied. We therefore conducted a cross-sectional study to evaluate coronary vascular function related to SUA levels in patients referred for stress myocardial perfusion PET. In addition, we examined whether the relationship between SUA levels and coronary vascular function differed by presence of diabetes based on the close relationship between hyperuricemia, gout and diabetes.[13–17]

## Methods

### Study cohort

All patients clinically referred for stress myocardial perfusion PET at the Brigham and Women’s Hospital in Boston, Massachusetts, USA between January 2006 and March 2014 were eligible for inclusion. Of those, we selected patients with at least one SUA level measurement during the 180-day period before or after the PET test date. In patients with multiple PET tests during the study period, we included the study closest to the date of SUA level measurement. The study protocol was approved by the Institutional Review Board of the Brigham and Women’s Hospital which granted a waiver of informed consent.

### Definition of exposure and outcome

SUA level measured by enzymatic colorimetric assay in 180 days before or after the PET test was the exposure of interest. Hyperuricemia was defined as ≥7 mg/dl in men and ≥6 mg/dl in women.

The primary outcomes of interest were stress MBF and CFR quantified using PET. Absolute MBF in milliliter/minute/gram (ml/min/g) was computed from the dynamic rest and stress imaging series with commercially available software (Corridor4DM; INVIA Medical Imaging Solution, Ann Arbor, MI) and previously validated methods.[18,19] CFR was calculated as the ratio of absolute MBF at stress over rest for the entire left ventricle. CFR <2 is known to be associated with worse cardiovascular outcomes in a general referral population.[11]

### PET imaging

Following standard imaging protocols, patients were studied with a whole-body PET/computed tomography scanner (Discovery RX or STE LightSpeed 64, GE Healthcare, Milwaukee, WI) after at least 4 hours of fasting. Patients refrained from caffeine- and methylxanthine-containing substances and drugs for 24 hours before their scans. Briefly, at rest, radionuclide imaging was obtained with an intravenous bolus administration of ^13^N-ammonia or ^82^rubidium. Then, a standard intravenous infusion of a vasodilator (i.e., dipyridamole, adenosine, or regadenoson) or dobutamine was given for pharmacologic stress. At peak stress, a second dose of ^13^N-ammonia or ^82^rubidium was injected, and images were recorded in the same manner. MBF was measured during rest and peak stress with ^13^N-ammonia or ^82^rubidium as a perfusion tracer, as described previously.[18–21] Heart rate, blood pressure, and 12-lead ECG were recorded at baseline and every minute during and after pharmacological stress. Left ventricular ejection fraction at rest and stress were calculated from gated myocardial perfusion images with commercially available software. In addition, summed rest score, summed stress score, and summed difference score (stress minus rest) were computed, with higher scores reflecting larger areas of myocardial scar, scar plus ischemia, or ischemia, respectively; summed stress scores ≤3 are generally considered normal.[22–24]

### Covariates

We assessed a number of pre-defined variables potentially related to hyperuricemia or coronary vascular function based on patient interview and/or medical record data in the Partners Healthcare Research Patient Data Registry from the 180-day period immediately before or after the PET test date. These variables were: age and sex; body mass index; comorbidities including gout, hypertension, diabetes, smoking, coronary artery disease, heart failure, and dyslipidemia; medications including beta blockers, calcium channel blockers, nitrates, angiotensin-converting enzyme inhibitors, diuretics, gout-related medications (*i*.*e*., allopurinol, febuxostat and colchicine), and aspirin; and laboratory data including SUA, serum creatinine, LDL cholesterol and C-reactive protein levels.

### Statistical analysis

Patient characteristics were compared between the hyperuricemia and normouricemia groups. Statistical significance was assessed with Wilcoxon rank sum tests or two-sided t-tests for continuous variables and Fisher’s exact or chi^2^ tests for binary variables. Normality was assessed using a combination of the Shapiro-Wilk test and visual inspection of descriptive statistics and histograms. Because data were not normally distributed, we used natural log transformation of SUA levels, CFR and MBF in all regression models. Pearson correlation was checked between log SUA, log CFR and log stress MBF. As diabetes is a strong predictor of coronary vascular function and CAD,[25] we tested for an interaction between SUA (as a continuous variable) and diabetes (yes/no) on CFR or stress MBF. Neither interaction was statistically significant. For primary analysis, we used unadjusted and multivariable linear regression models to examine the association between SUA levels and coronary vascular function in the main cohort. Our final models were adjusted for age, sex, summed stress score (i.e., a strong indicator of myocardial scar and ischemia), serum creatinine, and presence of gout diagnosis. Because prior myocardial scar or ischemia is a major determinant of CFR, we conducted a sensitivity analysis in which we performed multivariable linear regression models only in patients with summed stress scores ≤3.[22–24] We also performed stratified analysis by the presence of diabetes. All analyses were performed using SAS 9.3 Statistical Software (SAS Institute Inc., Cary, NC).

## Results

### Cohort characteristics

We identified a total of 382 patients including 208 with hyperuricemia and 174 with normouricemia. Table 1 presents patient characteristics. Mean (SD) age was 68.4 (12.4) years and 36% were female. Mean (SD) SUA level was 7.2 (2.6) mg/dl and 29% had gout. Cardiovascular comorbidities were prevalent as 85% had hypertension, 27% CAD and 11% heart failure. Diabetes was present in 44%, and 23% had any use of allopurinol or febuxostat.

**Table 1:** Study cohort characteristics.

N	Overall	Hyperuricemia	Normouricemia	p-value
	382	208	174	
*Mean ± SD*, *median [IQR] or percentage*				
Uric acid level, mg/dl	7.2 ± 2.6 7.0 [5.4, 8.7]	9.0 ± 2.1 8.5 [7.4, 10.1]	5.1 ± 1.2 5.3 [4.5, 6.0]	**<0.001**
Age, years	68.4 ± 12.4 69.0 [60.0, 77.0]	68.9 ± 12.0 69.0 [60.0, 78.0]	67.8 ± 12.9 69.0 [60.0, 76.0]	0.398
Female	36	37	34	0.68
Body mass index, kg/m^2^	29.0 [25.0, 34.0]	29.0 [26.0, 36.0]	28.0 [24.0, 33.0]	0.08
***Comorbidities***				
Hypertension	85	91	78	**0.001**
Diabetes	44	50	37	**0.012**
Gout	29	39	18	**<0.001**
Smoking	6	6	7	0.641
Coronary artery disease	27	28	26	0.682
Heart failure	11	16	4	**<0.001**
Dyslipidemia	75	77	72	0.242
Serum creatinine, mg/dl ^a^	1.2 [0.9, 1.9]	1.4 [1.1, 2.1]	1.0 [0.8, 1.4]	**<0.001**
LDL level, mg/dl ^b^	79.0 [58.0, 97.0]	79.0 [57.0, 97.0]	78.0 [61.0, 100.0]	0.721
***Medications***				
Beta blockers	69	71	66	0.318
Calcium channel blockers	26	32	20	**0.008**
Nitrates	19	24	13	**0.008**
ACE inhibitors	40	39	42	0.519
Diuretics	47	59	33	**<0.001**
Aspirin	66	67	65	0.6
Statins and other lipid lowering drugs	71	74	68	0.205
Colchicine	8	13	3	**<0.001**
Xanthine oxidase inhibitors	23	30	14	**<0.001**
***Cardiac function***				
Heart rate at rest, per minute	70.0 [63.0, 80.0]	71.0 [63.0, 79.5]	69.0 [62.0, 80.0]	0.312
Systolic blood pressure at rest, mmHg	142.0 [127.0, 162.0]	142.0 [127.0, 163.0]	142.0 [125.0, 161.0]	0.638
LVEF at rest, %^c^	53.0 [40.0, 62.0]	50.0 [35.0, 62.0]	56.0 [45.0, 63.0]	**0.004**
LVEF at stress, %^d^	57.0 [44.0, 67.0]	55.0 [39.0, 64.0]	61.0 [47.0, 70.0]	**0.001**
Sum stress score	4.0 [0.0, 14.0]	6.0 [0.0, 16.0]	2.0 [0.0, 11.0]	**0.004**
Sum difference score	1.0 [0.0, 6.0]	2.0 [0.0, 6.0]	0.0 [0.0, 5.0]	0.155
Sum rest score	0.0 [0.0, 6.0]	0.0 [0.0, 7.0]	0.0 [0.0, 3.0]	**0.003**
Myocardial blood flow at rest, mL/min/g	1.0 [0.8, 1.2]	0.9 [0.7, 1.2]	1.0 [0.8, 1.3]	0.055
Myocardial blood flow at stress, mL/min/g	1.5 [1.1, 2.1]	1.4 [1.0, 2.0]	1.6 [1.2, 2.3]	**0.003**
Coronary flow reserve	1.6 [1.2, 2.0]	1.5 [1.2, 1.9]	1.6 [1.2, 2.1]	**0.04**

SD: standard deviation, LDL: low density lipoprotein, ACE: angiotensin converting enzyme, LVEF: left ventricle ejection fraction

P-values are for the comparisons between hyperuricemia and normouricemia.

^a^ Missing in <1% of the hyperuricemia group and 2% of the normouricemia group

^b^ Missing in 20% of the hyperuricemia group and 22% of the normouricemia group

^c^ Missing in 3% of the hyperuricemia group and 2% of the normouricemia group

^d^ Missing in 2% of the hyperuricemia group and 6% of the normouricemia group

The mean (SD) uric acid level in milligram per deciliter was 9.0 (2.1) for the hyperuricemia group and 5.1 (1.2) for the normouricemia group. Hypertension, diabetes, heart failure, gout, and use of calcium channel blockers, nitrates, xanthine oxidase inhibitors, and diuretics were more common in patients with hyperuricemia. The patients with hyperuricemia had impaired renal function with median [IQR] serum creatinine of 1.4 [1.1, 2.1] mg/dl whereas the median [IQR] serum creatinine was normal in the normouricemia group (1.0, [0.8, 1.4]). Median LDL level was similar between the groups.

### Coronary vascular function

The median [IQR] summed stress score was 4 [0.0, 14] for the overall cohort, 6.0 [0.0, 16.0] for the hyperuricemia, and 2.0 [0.0, 11] for the normouricemia group, indicating more prevalent myocardial damage in patients with hyperuricemia. 185 patients (48.4%) in the overall cohort had a normal summed stress score ≤3. Median [IQR] CFR was 1.6 [1.2, 2.0] and median [IQR] stress MBF was 1.5 [1.1, 2.1] ml/min/g in the overall cohort. The median CFR and stress MBF were lower (worse) in the hyperuricemia group versus the normouricemia group. There were weak negative correlations between SUA and CFR (r = -0.13, p = 0.015) and between SUA and MBF at stress (r = -0.24, p<0.001) in the unadjusted analysis (see S1 and S2 Figs). At rest, the heart rate and systolic blood pressure were similar between the hyperuricemia and normouricemia groups, but left ventricular ejection fraction was lower in patients with hyperuricemia (Table 1).

Table 2 presents the main results from the multivariable linear regression models. In the final multivariable linear regression model adjusting for age, sex, diabetes, summed stress score, serum creatinine and gout, SUA was associated with stress MBF (β = -0.14, p = 0.01), but not with CFR (β = -0.07, p = 0.14). In a sensitivity analysis limiting to 184 patients with a normal summed stress score (≤3), SUA was associated with both CFR (β = -0.14, p = 0.037) and stress MBF (β = -0.16, p = 0.048).

**Table 2:** Association between serum uric acid level and coronary vascular function*.

Outcome variable	Model adjustment	Regression coefficient (Standard error)	p-value
All patients (n = 382)			
Coronary flow reserve	None	-0.12 (0.05)	**0.015**
	Age and sex	-0.11 (0.05)	**0.022**
	Age, sex, diabetes, and SSS	-0.08 (0.05)	0.101
	Age, sex, diabetes, SSS, and Cr	-0.05 (0.05)	0.255
	Age, sex, diabetes, SSS, Cr and gout	-0.07 (0.05)	0.138
Myocardial blood flow at stress	None	-0.28 (0.06)	**<0.001**
	Age and sex	-0.22 (0.06)	**<0.001**
	Age, sex, diabetes, and SSS	-0.16 (0.05)	**0.002**
	Age, sex, diabetes, SSS, and Cr	-0.15 (0.05)	**0.007**
	Age, sex, diabetes, SSS, Cr and gout	-0.14 (0.05)	**0.01**
Patients with SSS≤3 (n = 184)			
Coronary flow reserve	None	-0.15 (0.06)	**0.023**
	Age and sex	-0.16 (0.07)	**0.016**
	Age, sex, diabetes	-0.16 (0.07)	**0.014**
	Age, sex, diabetes, and Cr	-0.13 (0.06)	0.054
	Age, sex, diabetes, Cr and gout	-0.14 (0.07)	**0.037**
Myocardial blood flow at stress	None	-0.23 (0.08)	**0.003**
	Age and sex	-0.19 (0.08)	**0.014**
	Age, sex, diabetes	-0.18 (0.08)	**0.019**
	Age, sex, diabetes, and Cr	-0.15 (0.08)	**0.048**
	Age, sex, diabetes, Cr and gout	-0.16 (0.08)	**0.048**

SSS: summed stress score, Cr: serum creatinine

*Log transformed values of serum uric acid level, coronary flow reserve, myocardial blood flow at stress, and serum creatinine were used

### Stratified analysis

Characteristics of the diabetic (n = 167) and non-diabetic (n = 215) subgroups are presented in Tables 3 and 4. The mean age (SD) was 67.1 (10.3) years in the diabetic subgroup and 69.4 (13.8) in the non-diabetic subgroup. Median [IQR] BMI was 32.0 [28.0, 38.0] kg/m^2^ in the diabetes and 27.0 [24.0, 31.0] kg/m^2^ in the non-diabetes. Overall, patients with diabetes had higher prevalence of comorbidities including 38% with gout versus 22% with gout in the non-diabetic subgroup. 41.9% of the diabetic group and 53.5% of the non-diabetic group had a normal summed stress score. Median stress MBF was lower in the diabetic group but median CFR was the same in both diabetic and non-diabetic subgroups. In patients with diabetes (see S1 and S2 Figs), SUA was not correlated with either CFR (r = -0.02, p = 0.80) or MBF at stress (r = -0.13, p = 0.10) even in the unadjusted analysis. No association was noted in multivariable linear regressions (Table 5**)**.

**Table 3:** Characteristics of patients with diabetes.

N	Overall	Hyperuricemia	Normouricemia	p-value
	167	103	64	
*Mean ± SD*, *median [IQR] or percentage*				
Uric acid level, mg/dl	7.6 ± 2.7 7.4 [5.6, 9.3]	9.2 ± 2.1 8.9 [7.6, 10.2]	5.1 ± 1.2 5.2 [4.4, 6.1]	**<0.001**
Age, years	67.1 ± 10.3 68.0 [60.0, 75.0]	68.4 ± 10.0 68.0 [61.0, 77.0]	65.0 ± 10.4 66.0 [59.0, 73.0]	**0.042**
Female	34	37	28	0.243
Body mass index, kg/m^2^	32.0 [28.0, 38.0]	33.0 [28.0, 39.0]	31.5 [27.5, 36.0]	0.169
***Comorbidities***				
Hypertension	90	94	84	**0.037**
Gout	38	47	25	**<0.001**
Smoking	4	4	5	1
Coronary artery disease	30	28	33	0.523
Heart failure	13	18	3	**0.004**
Dyslipidemia	79	80	78	0.819
Serum creatinine, mg/dl ^a^	1.3 [1.0, 1.9]	1.4 [1.1, 1.9]	1.1 [0.9, 2.0]	0.115
LDL level, mg/dl ^b^	79.0 [56.0, 98.0]	79.5 [57.0, 98.0]	76.0 [55.0, 95.0]	0.531
***Medications***				
Beta blockers	73	72	75	0.656
Calcium channel blockers	29	38	16	**0.002**
Nitrates	26	30	20	0.163
ACE inhibitors	47	43	53	0.19
Diuretics	56	68	36	**<0.001**
Aspirin	72	74	70	0.625
Statins and other lipid lowering drugs	75	75	75	0.972
Colchicine	13	19	3	**<0.001**
Xanthine oxidase inhibitors	29	36	19	**0.018**
***Cardiac function***				
Heart rate at rest, per minute	70.0 [63.0, 80.0]	71.0 [64.0, 80.0]	69.5 [63.0, 80.0]	0.567
Systolic blood pressure at rest, mmHg	143.0 [125.0, 170.0]	144.0 [130.0, 172.0]	139.0 [122.0, 164.0]	0.204
LVEF at rest, %^c^	51.0 [39.0, 62.0]	50.5 [35.0, 62.0]	52.0 [42.0, 60.0]	0.53
LVEF at stress, %^d^	54.0 [40.0, 66.0]	54.0 [39.0, 65.0]	55.0 [44.5, 70.0]	0.205
Sum stress score	6.0 [0.0, 15.0]	6.0 [0.0, 15.0]	4.5 [0.0, 15.0]	0.597
Sum difference score	2.0 [0.0, 7.0]	3.0 [0.0, 7.0]	2.0 [0.0, 8.0]	0.864
Sum rest score	0.0 [0.0, 6.0]	1.0 [0.0, 6.0]	0.0 [0.0, 7.0]	0.362
Myocardial blood flow at rest, mL/min/g	0.9 [0.7, 1.2]	0.9 [0.7, 1.2]	0.9 [0.8, 1.2]	0.291
Myocardial blood flow at stress, mL/min/g	1.4 [1.1, 2.1]	1.5 [1.0, 2.1]	1.4 [1.2, 1.8]	0.709
Coronary flow reserve	1.6 [1.2, 2.1]	1.5 [1.2, 2.1]	1.6 [1.2, 2.0]	0.683

SD: standard deviation, LDL: low density lipoprotein, ACE: angiotensin converting enzyme, LVEF: left ventricle ejection fraction

P-values are for the comparisons between hyperuricemia and normouricemia.

^a^ Missing in 0% of the hyperuricemia group and 2% of the normouricemia group

^b^ Missing in 17% of the hyperuricemia group and 17% of the normouricemia group

^c^ Missing in 5% of the hyperuricemia group and 2% of the normouricemia group

^d^ Missing in 1% of the hyperuricemia group and 6% of the normouricemia group

**Table 4:** Characteristics of patients without diabetes.

N	Overall	Hyperuricemia	Normouricemia	p-value
	215	105	110	
*Mean ± SD*, *median [IQR] or percentage*				
Uric acid level, mg/dl	6.9 ± 2.5 6.7 [5.3, 8.3]	8.9 ± 2.0 8.3 [7.3, 9.9]	5.1 ± 1.2 5.3 [4.6, 5.9]	**<0.001**
Age, years	69.4 ± 13.8 70.0 [60.0, 80.0]	69.4 ± 13.7 70.0 [58.0, 79.0]	69.4 ± 14.0 71.0 [60.0, 81.0]	0.999
Female	37	36	38	0.763
Body mass index, kg/m^2^	27.0 [24.0, 31.0]	27.0 [25.0, 31.0]	27.0 [24.0, 31.0]	0.554
***Comorbidities***				
Hypertension	81	88	74	**0.013**
Gout	22	31	14	**0.002**
Smoking	8	8	8	0.863
Coronary artery disease	25	28	22	0.343
Heart failure	9	14	5	**0.015**
Dyslipidemia	71	74	68	0.303
Serum creatinine, mg/dl ^a^	1.2 [0.9, 1.7]	1.4 [1.0, 2.2]	1.0 [0.8, 1.2]	**<0.001**
LDL level, mg/dl ^b^	79.5 [62.0, 95.5]	79.0 [58.0, 92.0]	80.0 [67.0, 101.0]	0.421
***Medications***				
Beta blockers	65	70	61	0.169
Calcium channel blockers	24	26	22	0.526
Nitrates	14	18	9	0.057
ACE inhibitors	36	35	36	0.934
Diuretics	40	50	31	**0.006**
Aspirin	61	61	61	0.938
Statins and other lipid lowering drugs	68	72	63	0.156
Colchicine	5	7	3	0.295
Xanthine oxidase inhibitors	17	24	11	**0.012**
***Cardiac function***				
Heart rate at rest, per minute	70.0 [62.0, 80.0]	71.0 [63.0, 79.0]	69.0 [61.0, 80.0]	0.403
Systolic blood pressure at rest, mmHg	142.0 [128.0, 158.0]	140.0 [125.0, 157.0]	143.0 [130.0, 158.0]	0.427
LVEF at rest, %^c^	55.0 [42.0, 63.0]	50.0 [34.0, 62.0]	57.0 [46.0, 64.0]	**0.003**
LVEF at stress, %^d^	59.0 [45.0, 68.0]	55.0 [39.0, 64.0]	63.0 [51.0, 70.0]	**0.002**
Sum stress score	2.0 [0.0, 13.0]	5.0 [0.0, 18.0]	0.0 [0.0, 7.0]	**0.004**
Sum difference score	0.0 [0.0, 5.0]	0.0 [0.0, 6.0]	0.0 [0.0, 4.0]	0.174
Sum rest score	0.0 [0.0, 4.0]	0.0 [0.0, 7.0]	0.0 [0.0, 2.0]	**0.004**
Myocardial blood flow at rest, mL/min/g	1.0 [0.8, 1.3]	0.9 [0.8, 1.2]	1.0 [0.8, 1.3]	0.166
Myocardial blood flow at stress, mL/min/g	1.6 [1.1, 2.1]	1.4 [1.1, 1.9]	1.8 [1.2, 2.5]	**<0.001**
Coronary flow reserve	1.6 [1.2, 1.9]	1.5 [1.2, 1.7]	1.7 [1.3, 2.2]	**0.002**

SD: standard deviation, LDL: low density lipoprotein, ACE: angiotensin converting enzyme, LVEF: left ventricle ejection fraction

P-values are for the comparisons between hyperuricemia and normouricemia.

^a^ Missing in 1% of the hyperuricemia group and 2% of the normouricemia group

^b^ Missing in 23% of the hyperuricemia group and 25% of the normouricemia group

^c^ Missing in 2% of the hyperuricemia group and 3% of the normouricemia group

^d^ Missing in 4% of the hyperuricemia group and 6% of the normouricemia group

**Table 5:** Association between serum uric acid level and coronary vascular function for patients stratified by diabetes*.

Outcome variable	Model adjustment	Regression coefficient (Standard error)	p-value
*With diabetes*			
**Coronary flow reserve**	None	-0.02 (0.08)	0.803
	Age and sex	-0.01 (0.08)	0.876
	Age, sex, SSS and Cr	0.01 (0.07)	0.856
	Age, sex, SSS, Cr and gout	-0.003 (0.08)	0.969
**Myocardial blood flow at stress**	None	-0.15 (0.09)	0.1
	Age and sex	-0.14 (0.09)	0.111
	Age, sex, SSS, and Cr	-0.11 (0.08)	0.187
	Age, sex, SSS, Cr and gout	-0.10 (0.08)	0.242
*Without diabetes*			
**Coronary flow reserve**	None	-0.21 (0.06)	**0.001**
	Age and sex	-0.20 (0.06)	**0.003**
	Age, sex, SSS, and Cr	-0.14 (0.07)	**0.038**
	Age, sex, SSS, Cr and gout	-0.15 (0.07)	**0.022**
**Myocardial blood flow at stress**	None	-0.37 (0.08)	**<0.001**
	Age and sex	-0.27 (0.08)	**<0.001**
	Age, sex, SSS and Cr	-0.19 (0.07)	**0.01**
	Age, sex, SSS, Cr and gout	-0.19 (0.07)	**0.01**

SSS: summed stress score, Cr: serum creatinine

*Log transformed values of serum uric acid level, coronary flow reserve, myocardial blood flow at stress, and serum creatinine were used.

However, among patients with no diabetes, SUA (see S1 and S2 Figs) had a negative correlation with both CFR (r = -0.22, p = 0.001) and stress MBF (r = -0.31, p<0.001) in the unadjusted analysis. In multivariable linear regression adjusting for age, sex, summed stress score (a measure of the extent of myocardial scar and ischemia), serum creatinine and diagnosis of gout, a higher SUA remained modestly associated with a lower CFR (β = -0.15, p = 0.02) and stress MBF (β = -0.19, p = 0.01).

## Discussion

The potential causal role of SUA on CAD and other cardiometabolic diseases has been under debate over the past few decades. While a number of epidemiologic studies showed positive associations between SUA and CAD or cardiovascular disease,[1–3] Mendelian randomization studies did not find a causal role of SUA in CAD, cardiovascular disease or diabetes.[26,27] In this cross-sectional study of 382 patients with a wide range of SUA levels, we demonstrated an inverse association between SUA and coronary vascular function in patients with no or minimal myocardial scar or ischemia (i.e., summed stress score ≤3) or those without diabetes, but no association among diabetic patients. The degree of association between SUA and coronary vascular function in non-diabetic patients was modest with a beta-coefficient of -0.15 for CFR and -0.19 for MBF in the multivariable linear regression models.

This present study provides one of the most comprehensive evaluations of myocardial perfusion and coronary vascular function in relation to SUA levels. We examined not only the overall relationship between SUA and coronary vascular function in patients with and without prior myocardial scar or ischemia, but also the relationship stratified by the presence of diabetes. Since not all patients with high SUA have gout, an independent risk factor for cardiovascular disease, our analysis was adjusted for the presence of gout. As a result, we noted a modest negative association between SUA and coronary vascular dysfunction in patients without overt CAD (i.e., normal summed stress score) and those without diabetes, but not in the diabetic group. The lack of a significant association in diabetic patients may be explained by the fact that the modest effect of SUA on CFR seen in non-diabetic patients is likely overshadowed by the known strong association of diabetes and associated metabolic abnormalities (hyperglycemia and insulin resistance) with coronary microvascular dysfunction.[25,28] In other words, even if SUA has a modest causal role in determining coronary vascular function, the effect of SUA on coronary vascular function in diabetic patients may be too subtle. The observed difference in the association of SUA with coronary vascular dysfunction by diabetes may be also related to the greater prevalence of other comorbid conditions and/or use of cardiovascular medications in diabetic patients versus non-diabetic patients, which can be directly or indirectly related to coronary vascular dysfunction. While our findings need to be confirmed, it is worth investigating the longitudinal effect of SUA on coronary vascular dysfunction in patients with and without diabetes separately.

Another potential explanation for the difference in the association of SUA with coronary vascular dysfunction by diabetes may be related to the difference in the severity of underlying CAD between the diabetic and non-diabetic groups in the study cohort. In the diabetic group, 41.9% had a normal summed stress score (i.e., ≤3) while 53% did in the non-diabetic group. As seen in the sensitivity analysis limited to those with a normal summed stress score (≤3), SUA unlikely has a role in determining coronary vascular function in patients with established myocardial damages even if SUA is causally related to CAD.

This study has limitations. First, as discussed earlier, this study is based at a single academic center, in which the study patients were referred for a PET test for a clinical reason. Thus, the results may not be generalizable to those with hyperuricemia and no clinical symptoms of CAD or subtle CAD. Second, as this is a cross-sectional study, the causal relationship between SUA levels and coronary vascular function cannot be determined. Some patients may maintain high SUA levels for a long time unless they are treated. However, to reduce exposure misclassification (i.e., SUA level) in the study cohort, we required all the SUA levels to be drawn within 6 months from the PET test. Third, while the final models were adjusted for several important predictors of CAD risk including age, sex, renal function, gout diagnosis, and a summed stress score (i.e., a marker of myocardial scar and ischemia), there may be residual confounding. Fourth, this study is the first and largest study that investigated an association between SUA and coronary vascular function using a cardiac PET, but further confirmation of our results is necessary in a larger and more generalizable setting.

In conclusion, this cross-sectional analysis showed a modest inverse association between high SUA levels and coronary vascular function in patients without diabetes after adjusting for age, sex, serum creatinine, gout diagnosis and the extent and severity of perfusion defects. Such association was not noted in patients with diabetes. While our results need to be confirmed in different settings, this present study suggests that the effect of hyperuricemia on coronary vascular function or CAD differs by diabetes status and may be more evident in patients without diabetes. Furthermore, this study highlights the need for future research on the association between the change in SUA and the change in coronary vascular function over time, particularly in patients without diabetes.

## Supporting information

S1 Fig Pearson correlations between serum uric acid levels and myocardial blood flow (MBF) at stress. (DOCX)

S2 Fig Pearson correlations between serum uric acid levels and coronary flow reserve (CFR). (DOCX)

## Abbreviations

CAD: coronary artery disease
CFR: coronary flow reserve
IQR: interquartile range
LDL: low density lipoprotein
MBF: myocardial blood flow
MI: myocardial infarction
PET: positron emission tomography
SSS: summed stress score
SUA: serum uric acid
