# Supplementary material for: Assessment of coronary vascular function with cardiac PET in relation to serum uric acid
Source: PLoS One. 2018 Feb 13;13(2):e0192788. doi: 10.1371/journal.pone.0192788 (PMC5811013; doi:10.1371/journal.pone.0192788)
Supplement: S2 Fig — (DOCX) [file pone.0192788.s002.docx]

**S2 Fig.** **Pearson correlations between serum uric acid levels and coronary flow reserve (CFR)**
